# Engineering photosynthesis, nature’s carbon capture machine

**DOI:** 10.1371/journal.pbio.3002183

**Published:** 2023-07-11

**Authors:** Megan L. Matthews

**Affiliations:** 1 Department of Civil and Environmental Engineering, University of Illinois at Urbana-Champaign, Urbana, Illinois, United States of America; 2 Carl R Woese Institute for Genomic Biology, University of Illinois at Urbana–Champaign, Urbana, Illinois, United States of America; University of California, Davis, UNITED STATES

## Abstract

How can we sustainably feed our growing population as the climate changes? This Perspective argues that by engineering photosynthesis to increase carbon capture, we can mitigate climate change and increase food production.

## Teaser

Mitigating climate change and sustainably feeding our growing population in the changing climate are 2 significant challenges facing the global community. Engineering photosynthesis, nature’s carbon capture machinery, can help us surmount these threats.

For thousands of years, humans have sought to increase plant productivity to create better and more abundant food. The Green Revolution in the second half of the 20th century saw us make significant improvements in crop yields through increased use of fertilizers, irrigation, and strategic breeding of crops to develop new high-yielding varieties. The impacts of this agricultural revolution were vast and included averting hunger for millions of people [1]. Since then, our population has continued to grow, passing 8 billion people in 2022, and is projected to peak at around 10.4 billion people in 2086 [2]. Traditional approaches that have been used to increase crop productivity are now plateauing. To feed this population, we need to develop new approaches to further increase crop productivity, especially in regions of the world where people do not currently have reliable access to food.

Photosynthesis, the process plants use to convert light energy into chemical energy, is responsible for nearly all life on Earth. Most terrestrial plants use the C3 photosynthetic pathway to harvest light and convert that solar radiation into chemical energy in the form of glucose. Yet, photosynthesis is not an efficient process, and most terrestrial plants only convert about 1% to 2% of the incoming light energy into chemical energy. As a comparison, commercially available solar panels convert around 20% of solar energy into electricity. For most of history, there has not been a need for plants to improve on this 1% to 2% efficiency. However, if we want to further increase crop productivity, we need to increase the amount carbon that is being assimilated by the crops in the first place. Improving the efficiency of photosynthesis would increase this source amount of assimilated carbon, which could then be transported to the yield or to a plant organ of interest.

Beyond increasing crop production for food, improving photosynthetic efficiency would capture more CO_2_ from the atmosphere that can be stored in plant biomass and eventually sequestered in the soil or used to create bioproducts [3]. Removing CO_2_ from the atmosphere is an important climate change mitigation strategy, and photosynthesis is a natural way of capturing this carbon. To improve photosynthetic efficiency for greater carbon capture, we need to be able to modify large modules or components of the photosynthesis machinery or even add new components all together [4]. These complex changes are difficult to achieve through evolution or breeding but can, and have been, accomplished through plant engineering.

Energy is lost at multiple steps throughout photosynthesis, preventing plants from more efficiently capturing carbon and converting solar energy to chemical energy. When it comes to absorbing solar radiation, light-harvesting antenna in the chloroplasts only absorb radiation that have wavelengths between 400 nm and 700 nm, which is roughly equivalent to the visible light spectrum. The wavelengths in this spectrum account for less than 50% of the total energy in solar radiation, and some of these wavelengths are reflected instead of absorbed, further decreasing the amount of solar radiation that plants can convert to chemical energy. Moreover, chloroplasts often experience fluctuating light throughout a day, transitioning through sun–shade and shade–sun conditions due to clouds and other leaves or plants moving with the wind. During these transitions, it can take several minutes for photosynthesis to return to maximal operation under the new light condition, which has been estimated to cost 10% to 40% of potential CO_2_ assimilation [5].

Photorespiration and the stomatal and mesophyll conductances, which determine how much CO_2_ reaches the chloroplasts, can also limit photosynthetic efficiency. Photorespiration occurs when O_2_ instead of CO_2_ binds to Rubisco, a key enzyme in the carbon fixation pathway, producing a different compound that must be recycled through the photorespiration pathway to recover back some of the carbon for photosynthesis. This process can reduce photosynthetic efficiency by 20% to 50% [6]. The amount of photorespiration that occurs is related to the ratio of CO_2_ to O_2_ in the chloroplast, where higher ratios of CO_2_ to O_2_ result in less photorespiration. This ratio is dependent on how easily CO_2_ can enter the leaf cells and chloroplasts, which is determined by leaf anatomy and the stomatal and mesophyll conductances. Variations from the C3 photosynthetic pathway have evolved independently in several plant species to avoid photorespiration (Box 1).

**Box 1. C3, C4, and CAM photosynthesis**

Photosynthesis comes in several different forms, with C3, C4, and crassulacean acid metabolism (CAM) pathways being the most common in plants. Approximately 85% of plants, including major crops like soybean, rice, and wheat, use the C3 photosynthetic pathway. In these plants, the photosynthetic reactions occur in the chloroplasts of leaf mesophyll cells. CO_2_ enters the leaf through stomatal pores before making its way into the chloroplasts. When these stomata open to take in CO_2_, water is also released from the leaves through transpiration. As such, plants need to balance CO_2_ uptake with water loss. Under drought conditions, plants will keep their stomata closed to prevent the loss of water. This also prevents CO_2_ from entering the mesophyll cells, leading to an increase in photorespiration, which occurs when Rubisco, a key enzyme in photosynthesis, binds with O_2_ instead of CO_2_.

In response to photorespiration, the C4 and CAM pathways have evolved independently in about 3% and 6% of flowering plant species, respectively [7]. C4 and CAM photosynthetic pathways use more energy to convert solar energy to glucose than the C3 pathway, but they have the benefit of concentrating CO_2_ around Rubisco, thereby increasing the efficiency of the carboxylation reaction as CO_2_ is no longer competing with O_2_ to bind to Rubisco. In C4 plants, like maize and sugarcane, CO_2_ enters the mesophyll cells where it is converted to a carbon intermediate that is transported from the mesophyll cells into another leaf cell, the bundle sheath. In the bundle sheath cells, the carbon intermediate is then converted back into CO_2_ and binds to Rubisco and the carbon fixation pathway proceeds. In CAM plants, like pineapple and aloe vera, the stomata only open during the night to prevent water loss. The CO_2_ that enters the mesophyll chloroplasts is then converted into an intermediate. During the day, the carbon intermediates are converted back to CO_2_, where they then bind with Rubisco, and, as with the C4 process, carbon fixation proceeds. Since C4 and CAM plants store CO_2_ as intermediates, they have more flexibility in when to open their stomata, allowing them to conserve water better than C3 plants. C4 and CAM photosynthetic pathways tend to be found in plants that are native to hot, arid environments where water loss can be a major problem.

The current state of photosynthesis (in)efficiency, combined with the number of avenues for its improvement, underscores the potential for engineering photosynthesis to increase carbon capture for food and storage. Scientists have already demonstrated in greenhouse and field experiments how we can increase photosynthesis through engineering different parts of the process, including increasing how fast photosynthetic machinery responds to fluctuating light [8,9], adding shorter and less energetically costly photorespiration recovery pathways [6], and manipulating photosynthetic enzymes to increase reaction rates [10]. In addition to these modifications, there are still a vast number of options for photosynthesis engineering that are being explored, from expanding the range of wavelengths that can be absorbed by the light-harvesting antenna [11] to introducing carboxysomes, carbon-concentrating compartments from blue-green algae, into C3 photosynthetic pathways [12].

Given the complexity of photosynthesis and the many different avenues towards engineering it for increased efficiency and carbon capture, mathematical models have been useful tools for identifying which strategies are promising for implementation [9]. Models will continue to be important resources to identify and evaluate different engineering strategies for increasing carbon capture at the field scale, across developmental stages, and under future climate scenarios. Models can also be used to investigate the predicted combined impact from stacking multiple engineered traits, such as increased light absorption and increased enzymatic reaction rates or less costly photorespiration.

Despite successful greenhouse and field experiments demonstrating that we can engineer plant photosynthesis to increase carbon assimilation, there remain gaps to translating these and future engineering strategies from research labs and fields into crops that can be planted by farmers around the world. For photosynthesis engineering to have a role in mitigating climate change and sustainably feeding our growing population, we need to bridge these regulatory, economic, social, and political gaps through specific translational grants, industry and foundation partnerships, and initiatives designed to accelerate the pipeline from discovery science to societal impact. We are living in a period of significant change and upheaval caused by our own actions and inactions. To address these challenges and meet the needs of the next century, we must innovate and implement beyond our traditional tools for increasing crop productivity. Engineering photosynthesis for improved efficiency and increased carbon capture, when used in conjunction with other scientific and societal advances, can help us solve at least 2 significant global challenges of the next century.
